# Current Insights on Lipid-Based Nanosystems 2023

**DOI:** 10.3390/ph16121700

**Published:** 2023-12-08

**Authors:** Ana Catarina Silva, João Nuno Moreira, José Manuel Sousa Lobo

**Affiliations:** 1 UCIBIO (Research Unit on Applied Molecular Biosciences), REQUIMTE (Rede de Química e Tecnologia), MEDTECH (Medicines and Healthcare Products), Laboratory of Pharmaceutical Technology, Department of Drug Sciences, Faculty of Pharmacy, University of Porto, 4050-313 Porto, Portugal; slobo@ff.up.pt; 2 Associate Laboratory i4HB-Institute for Health and Bioeconomy, Faculty of Pharmacy, University of Porto, 4050-313 Porto, Portugal; 3 FP-BHS (Biomedical and Health Sciences Research Unit), FP-I3ID (Instituto de Investigação, Inovação e Desenvolvimento), Faculty of Health Sciences, University Fernando Pessoa, 4249-004 Porto, Portugal; 4 CNC—Center for Neurosciences and Cell Biology, Center for Innovative Biomedicine and Biotechnology (CIBB), Faculty of Medicine (Polo 1), University of Coimbra, Rua Larga, 3004-504 Coimbra, Portugal; jmoreira@ff.uc.pt; 5 Faculty of Pharmacy, Univ Coimbra—University of Coimbra, CIBB, Pólo das Ciências da Saúde, Azinhaga de Santa Comba, 3000-548 Coimbra, Portugal

Among the different types of nanosystems that have been investigated for therapeutic use, lipid-based ones are the most explored, as they have advantages over non-lipid nanosystems, especially for improving the transport and efficacy of drugs through different routes of administration, such as ocular, cutaneous, intranasal, and intravenous [1,2,3,4,5,6,7,8,9,10,11,12,13,14,15,16,17,18].

The concept of lipid-based nanosystems is broad and includes solid lipid nanoparticles (SLN), nanostructured lipid carriers (NLC), cationic lipid nanoparticles, liposomes, exosomes, nanoemulsions, microemulsions, and self-nanoemulsifying systems. Studies have shown that these nanosystems are promising for improving the efficacy of lipophilic drugs or nucleic acids in different therapeutic applications, especially those that respond to unmet medical needs [2,4,19,20,21,22,23,24,25,26].

In the second edition of the Special Issue on lipid-based nanosystems, we notice that research in this field remains very active, as we published 16 works, including 10 research articles and 6 review articles, which were the following:

1. Aljuffali, I.A.; Anwer, M.K.; Ahmed, M.M.; Alalaiwe, A.; Aldawsari, M.F.; Fatima, F.; Jamil, S. Development of Gefitinib-Loaded Solid Lipid Nanoparticles for the Treatment of Breast Cancer: Physicochemical Evaluation, Stability, and Anticancer Activity in Breast Cancer (MCF-7) Cells. *Pharmaceuticals* **2023**, *16*, 1549. https://doi.org/10.3390/ph16111549.
2. Ocaña-Arakachi, K.; Martínez-Herculano, J.; Jurado, R.; Llaguno-Munive, M.; Garcia-Lopez, P. Pharmacokinetics and Anti-Tumor Efficacy of PEGylated Liposomes Co-Loaded with Cisplatin and Mifepristone. *Pharmaceuticals* **2023**, *16*, 1337. https://doi.org/10.3390/ph16101337.
3. Arif, S.T.; Khan, M.A.; Zaman, S.u.; Sarwar, H.S.; Raza, A.; Sarfraz, M.; Bin Jardan, Y.A.; Amin, M.U.; Sohail, M.F. Enhanced Antidepressant Activity of Nanostructured Lipid Carriers Containing Levosulpiride in Behavioral Despair Tests in Mice. *Pharmaceuticals* **2023**, *16*, 1220. https://doi.org/10.3390/ph16091220.
4. Tyagi, R.; Waheed, A.; Kumar, N.; Ahad, A.; Bin Jardan, Y.A.; Mujeeb, M.; Kumar, A.; Naved, T.; Madan, S. Formulation and Evaluation of Plumbagin-Loaded Niosomes for an Antidiabetic Study: Optimization and In Vitro Evaluation. *Pharmaceuticals* **2023**, *16*, 1169. https://doi.org/10.3390/ph16081169.
5. Ahalwat, S.; Bhatt, D.C.; Rohilla, S.; Jogpal, V.; Sharma, K.; Virmani, T.; Kumar, G.; Alhalmi, A.; Alqahtani, A.S.; Noman, O.M.; et al. Mannose-Functionalized Isoniazid-Loaded Nanostructured Lipid Carriers for Pulmonary Delivery: In Vitro Prospects and In Vivo Therapeutic Efficacy Assessment. *Pharmaceuticals* **2023**, *16*, 1108. https://doi.org/10.3390/ph16081108.
6. Peczek, S.H.; Tartari, A.P.S.; Zittlau, I.C.; Diedrich, C.; Machado, C.S.; Mainardes, R.M. Enhancing Oral Bioavailability and Brain Biodistribution of Perillyl Alcohol Using Nanostructured Lipid Carriers. *Pharmaceuticals* **2023**, *16*, 1055. https://doi.org/10.3390/ph16081055.
7. Satyanarayana, S.D.; Abu Lila, A.S.; Moin, A.; Moglad, E.H.; Khafagy, E.-S.; Alotaibi, H.F.; Obaidullah, A.J.; Charyulu, R.N. Ocular Delivery of Bimatoprost-Loaded Solid Lipid Nanoparticles for Effective Management of Glaucoma. *Pharmaceuticals* **2023**, *16*, 1001. https://doi.org/10.3390/ph16071001.
8. Garrós, N.; Bustos-Salgados, P.; Domènech, Ò.; Rodríguez-Lagunas, M.J.; Beirampour, N.; Mohammadi-Meyabadi, R.; Mallandrich, M.; Calpena, A.C.; Colom, H. Baricitinib Lipid-Based Nanosystems as a Topical Alternative for Atopic Dermatitis Treatment. *Pharmaceuticals* **2023**, *16*, 894. https://doi.org/10.3390/ph16060894.
9. Tong, Y.; Shi, W.; Zhang, Q.; Wang, J. Preparation, Characterization, and In Vivo Evaluation of Gentiopicroside-Phospholipid Complex (GTP-PC) and Its Self-Nanoemulsion Drug Delivery System (GTP-PC-SNEDDS). *Pharmaceuticals* **2023**, *16*, 99. https://doi.org/10.3390/ph16010099.
10. Rincón, M.; Espinoza, L.C.; Silva-Abreu, M.; Sosa, L.; Pesantez-Narvaez, J.; Abrego, G.; Calpena, A.C.; Mallandrich, M. Quality by Design of Pranoprofen Loaded Nanostructured Lipid Carriers and Their Ex Vivo Evaluation in Different Mucosae and Ocular Tissues. *Pharmaceuticals* **2022**, *15*, 1185. https://doi.org/10.3390/ph15101185.
11. Paiva, D.d.F.; Matos, A.P.d.S.; Garófalo, D.d.A.; do Nascimento, T.; Monteiro, M.S.d.S.d.B.; Santos-Oliveira, R.; Ricci-Junior, E. Use of Nanocarriers Containing Antitrypanosomal Drugs for the Treatment of Chagas Disease. *Pharmaceuticals* **2023**, *16*, 1163. https://doi.org/10.3390/ph16081163.
12. Korzun, T.; Moses, A.S.; Diba, P.; Sattler, A.L.; Taratula, O.R.; Sahay, G.; Taratula, O.; Marks, D.L. From Bench to Bedside: Implications of Lipid Nanoparticle Carrier Reactogenicity for Advancing Nucleic Acid Therapeutics. *Pharmaceuticals* **2023**, *16*, 1088. https://doi.org/10.3390/ph16081088.
13. Subhan, M.A.; Filipczak, N.; Torchilin, V.P. Advances with Lipid-Based Nanosystems for siRNA Delivery to Breast Cancers. *Pharmaceuticals* **2023**, *16*, 970. https://doi.org/10.3390/ph16070970.
14. Gugleva, V.; Andonova, V. Recent Progress of Solid Lipid Nanoparticles and Nanostructured Lipid Carriers as Ocular Drug Delivery Platforms. *Pharmaceuticals* **2023**, *16*, 474. https://doi.org/10.3390/ph16030474.
15. Richards, T.; Patel, H.; Patel, K.; Schanne, F. Endogenous Lipid Carriers—Bench-to-Bedside Roadblocks in Production and Drug Loading of Exosomes. *Pharmaceuticals* **2023**, *16*, 421. https://doi.org/10.3390/ph16030421.
16. Torres, J.; Costa, I.; Peixoto, A.F.; Silva, R.; Sousa Lobo, J.M.; Silva, A.C. Intranasal Lipid Nanoparticles Containing Bioactive Compounds Obtained from Marine Sources to Manage Neurodegenerative Diseases. *Pharmaceuticals* **2023**, *16*, 311. https://doi.org/10.3390/ph16020311.

Among the articles published in this Special Issue on lipid-based nanosystems, the lipid nanoparticles, specifically NLC, are the most explored, which suggests the potential of these systems to reach clinic in the upcoming years. Regarding the most investigated diseases, these include cancer, brain diseases, ocular diseases, skin diseases, microbial infections, and metabolic diseases. Thereby, we hope that all these works will contribute to the advancement of these scientific fields.

We would like to thank all the authors of this Special Issue for contributing high-quality articles, as well as all the reviewers who critically evaluated these works. We also thank to the assistant editor Evelyn Du for her kind help in managing this Special Issue.
